# Meeting of Chicago Medical Society

**Published:** 1863-09

**Authors:** 


### MEETING OF CHICAGO MEDICAL SOCIETY,
August 7th, 1863.

#### HYDROCELE,—SUPPURATIVE DIATHESIS; SULPHITES, &c.

Reports of cases being in order, Dr. WANZER related a case of hydrocele in a man 60 years of age. During the past winter and spring he had tapped the hydrocele twice, the first time drawing off a quart of limpid serum, and the second time but little more than half that quantity. The fluid, however, again slowly accumulated and distended the sac to a degree very burdensome to the patient.

On consultation with Dr. FISHER, an operation for radical cure was determined upon. Accordingly, Dr. FISHER performed the operation by an incision, between four and five inches in length, laying the sac of the hydrocele freely open. About one pint of slightly turbid serum escaped. The testicle appeared healthy; and the wound was dressed with lint.

When the suppurative process was established, Dr. WANZER said, the pus was "sanious and most horribly offensive to the smell, and evident symptoms of phlebitis were present. There was general anemia and palor of countenance, approaching the hippocratic expression." He used disenfectant washes to the wound, and gave, internally, the muriated tincture of iron, freely. No improvement took place, and Dr. FISHER was again called in consultation. In accordance with his advice, the patient was given one drachm doses of *sulphite* of lime, every four hours. The same local applications were continued as before. When eight doses of the medicine had been taken, a marked change for the better was observable in all the symptoms. The remedy was continued at the same rate until one ounce more had been taken, when the improvement in the symptoms, both general and local, was such as to render any further medication unnecessary.

The wound rapidly cicatrized; and the patient has pursued his usual occupation (gardener,) several months since. In concluding the case, Dr. WANZER, said, "there was a mysterious something that rapidly and decidedly changed the diathesis of the patient in less than forty-eight hours. If it was the sulphite of lime, will some member of the Society explain its mysterious action?"

Dr. N. S. DAVIS, inquired whether the free incisions into the sac, exposing its whole internal surface, and the consequent risk of severe inflammation and unhealthy suppuration, was necessary to effect the radical cure of hydrocele? He related a case of hydrocele in a young man, who recently came under his care, in which a cure was speedily effected without the occurrence of any severe symptoms, by means of the seton, as recommended by Dr. GROSS, in his large work on surgery. It seemed to him that the seton was a much more safe and equally certain means of obtaining a radical cure.

Dr. I. HATCH remarked, that he had used the seton, and seen it used by others, for the permanent cure of hydrocele for several years past, and with uniform success. He regarded it as preferable to any other method. He said, the fluid in the sac escaped gradually by the silken threads of the seton, and its place was speedily supplied by plastic lymph, resulting in complete obliteration of the sac. The threads of the seton should be removed as soon as the plastic effusion is sufficiently copious to render the scrotal tumor solid instead of fluctuating.

Dr. E. L. HOLMES wished to ask, in connection with the subject of suppurative diathesis suggested by the case of Dr. WANZER, what is the pathology of those cases which come to the physician not unfrequently, in which the patient, in apparently good general health, complains that his "blood is bad," because every slight wound he gets festers or suppurates and makes a sore instead of healing up kindly; and sometimes without any scratch or injury, pustules of impetigo, ferunculi, or malignant pustules make their appearance, on some part of the cutaneous surface. Such patients were familiar to every practitioner, and he would like to hear some explanation of their pathology, with a view to some rational treatment.

Dr. M. O. HEYDOCK remarked, that the questions asked by Drs. HOLMES and WANZER involved investigations of the highest interest and importance. The actual pathological condition of the blood in those cases presenting a tendency to suppuration, pyæmia, erysipelas, etc., is a subject now undergoing active investigation, both in this country and Europe. The experiments of Dr. POLLI, in relation to the action of the sulphites in counteracting blood-poisoning, and their subsequent use by members of this Society, in the successful treatment of malignant erysipelas and kindred affections, certainly opened a new field of study in rational therapeutics. The new and more extensive application of bromine in the treatment of hospital gangrene, as illustrated by the Reports from the Military Hospitals of Louisville and elsewhere, belongs to the same field of inquiry. Still, he was not prepared to attempt a full answer to the questions proposed by either of the gentlemen who had preceded him in this discussion.

Dr. N. S. DAVIS, suggested that the only explanation which could be given in relation to the *modus operandi* of the sulphites, was that afforded by the experiments of Dr. POLLI, together with their well-known efficacy in preventing fermentation in inanimate matter. That the minute atom of infectious or contagious poison that gains access to the blood, whether by inhalation or inoculation, multiplies itself until it becomes sufficient to disturb the whole system, by a process analogous to that of fermentation, there can scarcely be a doubt; and, if so, we could see no reason why the presence of a sufficient quantity of the sulphites in the blood would not interrupt that process as certainly as it does in inanimate compounds out of the living system. Whether acting upon the clear and important idea here set forth, other remedies may not be discovered more efficacious than the sulphites remains to be determined. He suggested a distinction between the action of the sulphites and the class of remedies having chlorine, bromine, iodine, etc., as a part of their composition. The latter produce their effects by destroying the noxious gases and agents with which they come in actual contact, and by moderately promoting the action of the excretory organs of the system. Hence, they are properly called *disenfectants* and *alterants.*

On the other hand, the presence of the sulphites prevents those changes in animal and vegetable substances, by which noxious agents are evolved; and, hence, are properly called *anti-septics.* The distinction he deemed an important one in practice. In relation to the cases of "bad blood," or "impure blood," alluded to by Dr. HOLMES, he suggested that they were capable of being arranged into three classes. The first, are such cases as arise from some disorder of the excretory actions, such as habitual deficiency in the eliminations from the skin, kidneys, or liver, thereby leaving the effete materials of the system in excess in the blood. These cases are most apt to occur during the transition of the seasons, the spring and autumn, but may occur at any period of the year in persons of sedentary habits, or in those whose avocations habitually disturb the natural equilibrium of action throughout the animal economy. The second class of cases originate from a disturbed or imperfect metamorphosis of tissues. They are seen most frequently during recovery from attacks of the typhoidal class of acute diseases. They are seen frequently also among those who live in damp and poorly ventilated apartments, and in those who habitually retard metamorphosis by the use of alcoholic drinks.

The third class of cases arise from the positive reception into the system of some of the milder class of septic poisons. These are much less numerous than the preceding classes, but not less important. The mere statement of the pathology of these several classes of what might be called minor blood maladies, suggests their proper remedial management.

